# Tuning the Electronic Conductivity in Hydrothermally Grown Rutile TiO_2_ Nanowires: Effect of Heat Treatment in Different Environments

**DOI:** 10.3390/nano7100289

**Published:** 2017-09-23

**Authors:** Alena Folger, Julian Kalb, Lukas Schmidt-Mende, Christina Scheu

**Affiliations:** 1 Max-Planck-Institut für Eisenforschung GmbH, Max-Planck-Str. 1, 40237 Düsseldorf, Germany; a.folger@mpie.de; 2 Department of Physics, University of Konstanz, POB 680, 78457 Konstanz, Germany; julian.kalb@uni-konstanz.de (J.K.); lukas.schmidt-mende@uni-konstanz.de (L.S.-M.); 3 Materials Analytics, RWTH Aachen University, Kopernikusstr. 10, 52074 Aachen, Germany

**Keywords:** black TiO_2_, nanowire, conductivity, electron energy loss spectroscopy, oxygen vacancy, defects

## Abstract

Hydrothermally grown rutile TiO_2_ nanowires are intrinsically full of lattice defects, especially oxygen vacancies. These vacancies have a significant influence on the structural and electronic properties of the nanowires. In this study, we report a post-growth heat treatment in different environments that allows control of the distribution of these defects inside the nanowire, and thus gives direct access to tuning of the properties of rutile TiO_2_ nanowires. A detailed transmission electron microscopy study is used to analyze the structural changes inside the nanowires which are correlated to the measured optical and electrical properties. The highly defective as-grown nanowire arrays have a white appearance and show typical semiconducting properties with n-type conductivity, which is related to the high density of oxygen vacancies. Heat treatment in air atmosphere leads to a vacancy condensation and results in nanowires which possess insulating properties, whereas heat treatment in N_2_ atmosphere leads to nanowire arrays that appear black and show almost metal-like conductivity. We link this high conductivity to a TiO_2−*x*_ shell which forms during the annealing process due to the slightly reducing N_2_ environment.

## 1. Introduction

Nanostructured titanium dioxide (TiO_2_) is a promising material in the field of energy conversion and storage [1]. In most TiO_2_ applications, the efficiency of the device is determined by three consecutive processes: light absorption, charge separation, and electron transport. Although TiO_2_ is widely used for energy applications, the efficiency of bare TiO_2_ is limited by a wide band gap of around 3 eV [2] and a relatively low electron conductivity [3,4]. To overcome these limitations, defect engineering can be used to optimize the optical band gap and the electrical properties. In combination with an optimized geometry, which can be derived from theoretical calculations [5], defect engineering enables the fabrication of highly active devices.

Defects can be introduced in TiO_2_ by metal [6] and nonmetal [7,8] impurities or dopants. However, this approach has the drawback that the dopants, especially d-block transition metals, also act as recombination centers for the generated electron hole pairs [9], which in turn lowers the efficiency of the device. Other approaches to produce defective TiO_2_ without doping are mediated by the incorporation of Ti^3+^ and oxygen vacancies (O_vac_) via reduction [10,11,12], which might introduce surface disorder in addition [13,14,15]. Most approaches use hydrogen environment and elevated temperatures [14,16,17] or a hydrogen plasma [18,19] to produce defective TiO_2_. Similar types of defective TiO_2_ can be obtained if active metals, such as Zn [10,20], Al [21], or Mg [22], are used as a reductant. However, these harsh reductive conditions are not mandatory to obtain defective TiO_2_. Instead, black TiO_2_ nanoparticles with surface disorder can be obtained by annealing amorphous nanoparticles in Ar gas [13]. Concededly, there are no reports which show that crystalline TiO_2_, e.g., rutile TiO_2_ nanowires (NWs), can be reduced in an oxygen-deficient atmosphere, such as vacuum, Ar, or N_2_.

The effect of defect engineering on the optical band gap and the apparent color, which can be tuned from yellow over blue to black, has been studied extensively [16,23,24]. Apart from that, reports about how structural changes, such as the introduction of O_vac_ or surface disorder, influence the electrical properties are rare. So far, Nowotny and co-workers studied the influence of defect disorder on the semiconducting properties of rutile TiO_2_ and found a strong effect on the electrical properties [25,26,27]. Especially, a high amount of O_vac_, which is intrinsically found in rutile TiO_2_, leads to strong n-type characteristics. In addition, the influence of O_vac_ on the conducting properties of TiO_2_ NWs was evaluated by intensity-modulated photocurrent spectroscopy. For oxygen-deficient NWs, two electron-transport modes, a trap-free mode in the core and a trap-limited mode near the surface, were detected [28]. Recently, Lü et al. [29] investigated the effect of the surface disorder on the electrical properties. On a 40 nm thick bilayer structure of crystalline anatase (≈20 nm) and amorphous TiO_2_ (20 nm), which serves as a model system, they found a metallic conductivity at the interface between the crystalline and the amorphous part. These results give a first hint on the electrical properties of the defective, black TiO_2_. Admittedly, in this model system the amorphous layer does not represent the surface disorder found in black TiO_2_ adequately. It is much thicker and does not show any ordering phenomena [29].

In this work, we present how the electrical properties of TiO_2_ NW arrays, incorporating rutile TiO_2_ NWs with different defect states, can be changed. A detailed analysis of the nanostructure and the local chemical environment of three differently treated NW arrays, in combination with our results from ultraviolet-visible (UV–Vis) and current-voltage (IV) measurements, leads to a better understanding of the underlying mechanism that are responsible for the electronic properties of defective TiO_2_. The results show how TiO_2_ NWs, which are intrinsically n-type semiconductors in the as-grown state, can be converted to almost insulating TiO_2_ NWs or NWs with a metal-like conductivity simply by using an appropriate atmosphere for the post-growth annealing.

## 2. Results

### 2.1. (Internal) Nanostructure and Local Chemical Environment

**Figure 1:**
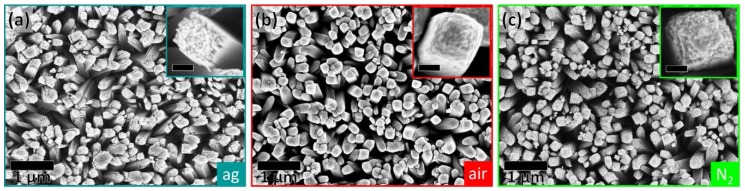
Scanning electron microscopy (SEM) images of nanowire (NW) arrays, which are (**a**) as-grown (ag), (**b**) annealed in air, and (**c**) annealed in N_2_. The insets show a high magnification SEM image of a single NW from the respective NW array. The scale bar of the inset is 50 nm.

Scanning electron microscopy (SEM) investigations (Figure 1) reveal that the NW arrays of the three samples consist of NWs which grow almost perpendicular to the fluorine tin oxide (FTO) substrate and are of similar size (diameter of as-grown: 164 ± 31 nm, annealed in air: 172 ± 16 nm, annealed in N_2_: 157 ± 28 nm). The high magnification SEM images in the insets of Figure 1 disclose slight morphological changes at the tip of the NWs. The as-grown NWs (Figure 1a) possess a rough tip, which is built by a bundle of nanofibers, as shown before by Wisnet et al. [30]. This structure is removed for the NWs annealed in air (Figure 1b), which have a much smoother surface. The tip of the NWs annealed in N_2_ looks like an intermediate state between the as-grown NWs and the NWs annealed in air, although it was annealed at the same temperature for the same time. For the NWs annealed in N_2_, the nanofiber bundle is still visible at the tip (Figure 1c), but not as prevalent as in the as-grown NWs.

**Figure 2:**
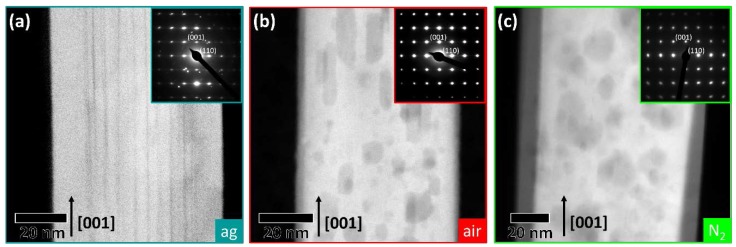
High-angle annular dark-field scanning transmission electron microscopy (STEM) image and a corresponding electron diffraction pattern (inset) for (**a**) an as-grown NW, (**b**) a NW annealed in air, and (**c**) a NW annealed in N_2_. All images show a representative area in the center of its respective NW and the diffraction patterns are taken from entire NWs.

The high-angle annular dark-field scanning transmission electron microscopy ((S)TEM) images in Figure 2, all taken from the central part of appropriate NWs, show more significant changes inside the NWs due to the annealing. While the as-grown NW is built by a bundle of nanofibers, as indicated from the SEM image, the annealed NWs are a single-crystalline material, which is interspersed with voids [31,32]. Nevertheless, SEM showed that even for the annealed NWs there are still residuals of the former nanofiber bundle at the tip (Figure 1b,c). The NW annealed in air does not show any further changes besides the voids, whereas the NW annealed in N_2_ has internal voids and in addition a distinct core-shell like structure with an approximately 10 nm thick shell. A similar shell can be detected for as-grown NWs and NWs annealed in air but it is only 1–3 nm thick. Although the nanostructures of the three NWs differ, no changes in crystallography can be detected. The diffraction patterns in the insets of Figure 2 correspond to rutile TiO_2_ acquired in the $\left[1\bar{1}0\right]$ zone axis and deviate only by the streaking in the diffraction peaks in [110] direction, which is visible for the as-grown NW. This streaking arises from the nanofiber bundle and the high defect density in the as-grown NW [30]. Thus, neither the heat treatment in air nor in N_2_ leads to a phase transformation.

**Figure 3:**
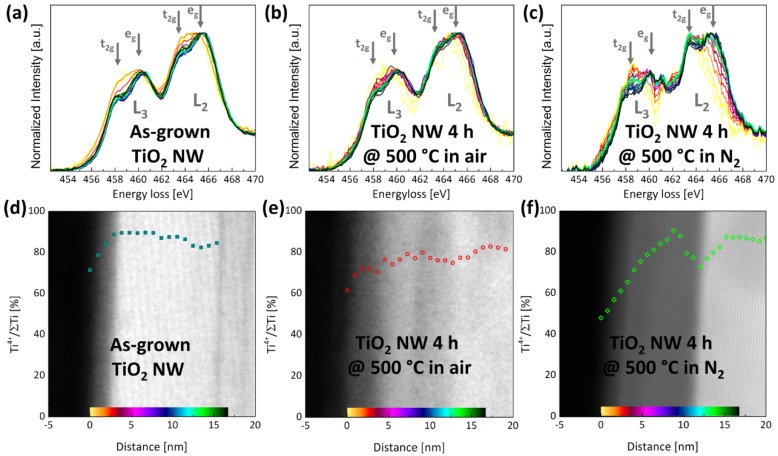
(**a**–**c**) Position resolved electron energy loss (EEL) spectra of the Ti-L_2,3_ edge, for (**a**) an as-grown NW, (**b**) a NW annealed in air, and (**c**) a NW annealed in N_2_. The positions of the spectra are marked in the STEM images of (**d**–**f**) with a specific color, which is the same for the respective Ti-L_2,3_ edge (the color changes from the NW surface to the center (left to right) in the following order: yellow, orange, red, pink, purple, blue, cyan, green, black). In (**d**–**f**), the Ti^4+^ gradient is overlaid with the STEM image.

Despite the changes in the nanostructure, there are also differences in the local chemical environment of the three different NWs close to the surface. Figure 3a–c shows electron energy-loss (EEL) spectra of the Ti-L_2,3_ edge with different distances to the surface. Close to the surface (yellow lines), the Ti-L_2,3_ edge is shifted to lower energies by around 1 eV and the energy loss near edge fine structure (ELNES) shows that the splitting of the L_2_ and L_3_ peaks into a doublet is not resolved. This *t_2g_-e_g_* splitting is typical for rutile TiO_2_ and results from a distorted octahedral surrounding of Ti by oxygen ions [33], but cannot be detected for Ti close to the surface. Instead, the Ti-L_2,3_-edge is formed by broad peaks. Depending on the heat treatment, the typical ELNES of rutile TiO_2_ occurs closer or more far away from the surface. For the NWs annealed in air, the ELNES shows the typical shape of rutile TiO_2_ with a pronounced *t_2g_-e_g_* splitting after moving 1.8 nm towards the center (orange line in Figure 3b). For the as-grown NW, the broad L_2_ and L_3_ peaks in the ELNES are observed in the first 2.9 nm of the surface region (red line in Figure 3b). The NW annealed in N_2_ has the largest region (up to 4.8 nm, dark red line in Figure 3c), where one can find an ELNES without pronounced *t_2g_-e_g_* splitting. Moving farer away from the surface, the ELNES of the as-grown NW and the NW annealed in air does not change anymore, but for the NW annealed in N_2_ one can see that 10.4 nm away from the surface (light cyan line in Figure 3c), the ELNES changes again. Following the method described by Stroyanov et al. [34], the Ti-L_2,3_ edge is used to calculate the amount of Ti^4+^ relative to the total amount of Ti, which is mainly a sum of Ti^4+^ and Ti^3+^. Figure 3d–f are overlays of the resulting Ti^4+^ gradients with a STEM image of the analyzed NW area. The shift of the Ti-L_2,3_ edge towards lower energies close to the surface is related to a lower amount of Ti^4+^ in this area. Thus, close to the surface, the NWs are not fully oxidized. Inside the NW, the as-grown NW and the NW annealed in air have a constant amount of Ti^4+^ of around 80%. For the NW annealed in N_2_, the changes of the ELNES around 10.4 nm are also linked to a lower amount of Ti^4+^ and the overlay in Figure 3f shows that this decrease of Ti^4+^ is closely related to the core-shell interface. The lack of Ti^4+^ results in an off-stoichiometric TiO_2−*x*_. In the following, the shell material will be denoted as TiO_2−*x*_ to account for the high oxygen deficiency.

**Figure 4:**
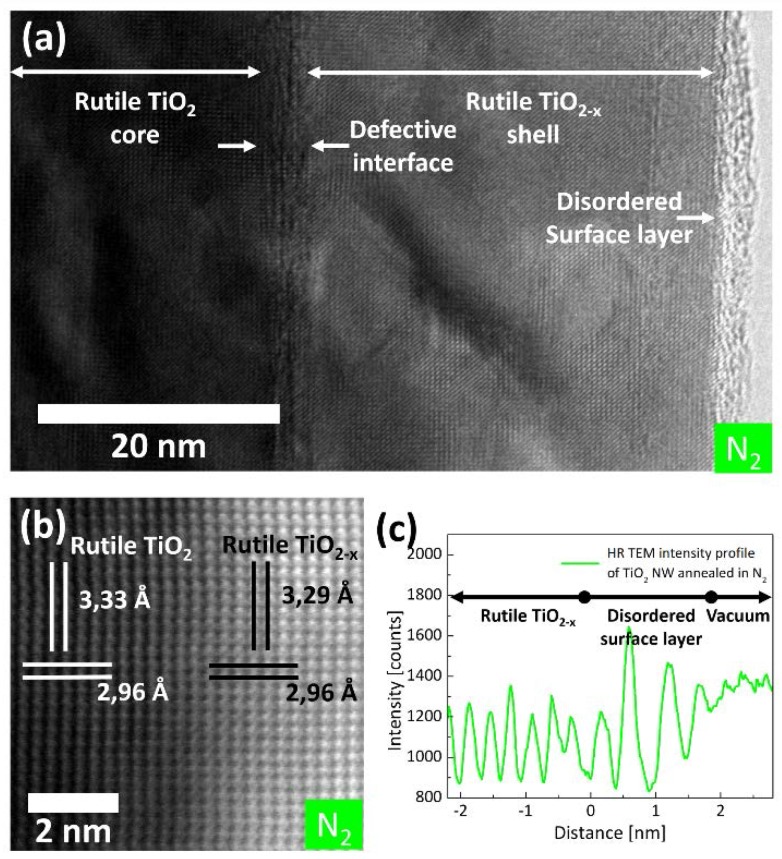
(**a**) High resolution (HR) transmission electron microscopy (TEM) image of a NW annealed in N_2_ showing the surface near region. (**b**) Annular bright field STEM image of the interface between the rutile TiO_2_ core and the TiO_2−*x*_ shell. (**c**) Intensity line scan of (**a**) showing an out-of-plane periodicity in the disordered surface layer parallel to the [001] growth direction.

To study the core-shell structure in more detail, Figure 4a shows a high resolution (HR) TEM image of a NW annealed in N_2_. This NW has a comparable thick shell to facilitate the analysis. One can see that the NW consists not only of a core and a shell, but of four distinctive areas. The rutile TiO_2_ core and the crystalline TiO_2−*x*_ shell are separated by a defective interface area and the shell is covered with a disordered surface layer. Around 80% of the NW volume can be assigned to the core, which is rutile. The shell is also crystalline and covers around 20% of the NW volume. The high resolution annular bright-field STEM image in Figure 4b shows no differences in the crystal structure of the rutile core and the shell, except a small change in the *d*-spacing between {110} planes (core: *d*_110_ = 3.33 Å, shell: *d*_110_ = 3.29 Å). Although the electron energy-loss spectroscopy (EELS) analysis shows that the shell consists of off-stoichiometric TiO_2−*x*_, no inhomogeneity in the oxygen distribution can be detected in the annular bright field STEM image (Figure 4b). Thus, an ordering of a significant amount of O_vac_ in this part of the NW is unlikely, as it would lead to periodic changes in the atomic columns, which should be visible in annular bright-field STEM. However, in all imaging conditions, this shell appears in a different contrast compared to the core. In the shell area, a sample thickness of 110 nm is derived using the low-loss EEL spectrum and assuming an inelastic mean free path of 276 nm for rutile TiO_2_ [35]. Considering an error of around 10% for the thickness determination by EELS [36], this thickness estimation is in good agreement with the total thickness of the analyzed NW, which is also shown in Figure 2c (around 100 nm). Thus, the changes in contrast cannot be related to a thickness effect but might be related to a change in the density of the material. The contrast changes might also be affected by the incorporation of nitrogen, but EELS measurements in the shell area show no incorporation of nitrogen within our detection limits of ≈1 at%. It is noteworthy that this observation cannot be confirmed by methods other than EELS with high lateral resolution because the nitrogen and the titanium signal overlap in other spectroscopic techniques, such as Auger and wavelength dispersive X-ray spectroscopy. The defective area, which can be seen in the HR TEM image of Figure 4a between the TiO_2_ core and the TiO_2−*x*_ shell is around 1.9 ± 0.3 nm thick. The disordered surface layer of the NW has a thickness of 2.2 ± 0.3 nm and is not completely amorphous, but shows some periodicity perpendicular to the [001] direction. Figure 4c is an intensity profile of Figure 4a in the first 4 nm next to the vacuum and perpendicular to the NW surface. This profile shows two periodic areas, but with different periodicity. The periodicity of the TiO_2−*x*_ shell corresponds to the lattice spacing of {110} planes in rutile TiO_2_. Closer to the vacuum, there is a second material, which is also periodic to a certain extent, but the related lattice distances are much bigger (≈5 Å). This in-plane ordering in an amorphous phase is due to the underlying substrate periodicity and has been observed for other systems as well [37].

### 2.2. Optical and Electrical Properties

**Figure 5:**
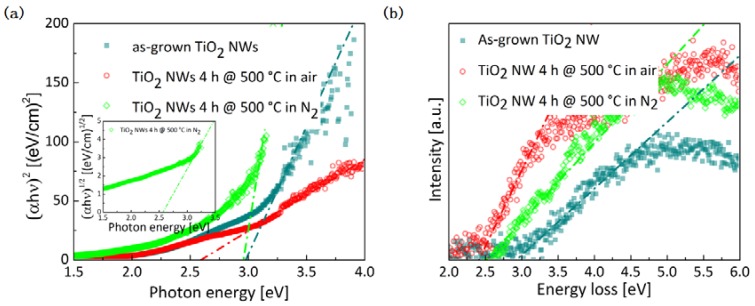
(**a**) Tauc plot for direct band gap and (**b**) zero-loss subtracted low-loss EEL spectra for NWs which are as-grown (petrol squares), annealed in air (red circles), and annealed in N_2_ (green lozenges). The inset in (**a**) shows the Tauc plot for an indirect band gap for the NW array annealed in N_2_.

The changes in the internal nanostructure, which are induced by annealing in different environments, influence the optical properties of the NWs. While the NW arrays incorporating as-grown NWs or NWs annealed in air appear white, the NW arrays annealed in N_2_ are black. This color change indicates more light absorption in the visible range for the NWs annealed in N_2_. Figure 5a shows Tauc plots for direct allowed band gap transitions of the three different NW arrays. The as-grown NWs have a band gap of 2.98 ± 0.06 eV, which is in good agreement with previous measurements [32]. The band gap of NWs annealed in air is significantly reduced to 2.59 ± 0.04 eV, but for the NWs annealed in N_2_, the obtained direct band gap is again 2.96 ± 0.03 eV. In addition, there is an indirect transition for the NWs annealed in N_2_ with an indirect band gap of around 2.57 ± 0.02 eV (inset in Figure 5a). In contrast, no strong indirect transition can be detected for the as-grown NWs and the NWs annealed in air. Absorption spectra allow not only the determination of the band gap but are also suitable to measure the so-called Urbach energy, which is a measure of the disorder in materials and leads to additional states within the band gap [38]. The Urbach energy of the as-grown NWs and the NWs annealed in air and in N_2_ is 0.61 ± 0.01 eV, 0.55 ± 0.01 eV, and 1.65 ± 0.01 eV, respectively. Since UV–Vis can only probe the band gap on a large scale and as the results might be influenced by the periodicity of the NW array, and the resulting interference effects, additional band gap measurements were performed using EELS. Figure 5b shows the corresponding zero-loss subtracted low-loss EEL spectra of the different NWs. The band gap values derived from the EELS measurements (as-grown: 2.93 ± 0.12 eV, annealed in air 2.41 ± 0.06 eV, annealed in N_2_ 2.66 ± 0.14 eV) are in good agreement with the values obtained by UV-Vis, considering the indirect transition for the NW annealed in N_2_.

**Figure 6:**
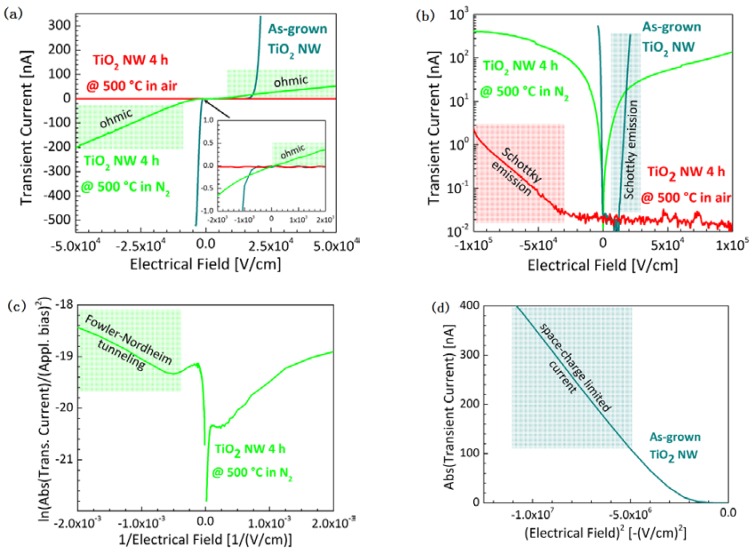
Transient current characteristics through as-grown NWs, NWs annealed in air, or in N_2_ that is measured between a PtIr top and an FTO bottom electrode. The different plots emphasize several conduction-limiting mechanisms: (**a**) Linear plot showing ohmic behavior and the inset is a zoom in on the point of origin, (**b**) Schottky plot, (**c**) Fowler–Nordheim plot and (**d**) space–charge-limited current plot [39].

Besides the optical properties of the NWs, the electronic properties are affected by the heat treatments. Figure 6a shows the IV-characteristics of the as-grown NWs, the NWs annealed in air, and the NWs annealed in N_2_. Significant differences in the electronic properties of the three devices regarding the conduction limiting mechanisms can be observed.

The as-grown NWs block the transient current for electrical fields between 0 and 12 kV/cm (petrol line, Figure 6a). At higher electrical fields, the transient current is increasing exponentially and is hence affected by Schottky emission (petrol line, Figure 6b). For an increasing negative bias, the IV-characteristic turns quickly from an exponential increase into an increase that is proportional to the squared electric field arising from a space-charge-limited current (Figure 6d) [39]. Thus, the Schottky barrier at the PtIr/TiO_2_ interface is smaller than the one at the FTO/TiO_2_ interface.

TiO_2_ NWs annealed in air block the transient current for electrical fields between −25 kV/cm and at least 100 kV/cm (red line, Figure 6a,b), which corresponds to the highest applicable bias in the employed setup. The IV-characteristics of the PtIr/TiO_2_ interface become completely exponential and hence the transient current is limited by a Schottky emission across the whole measured bias range (red line, Figure 6b) [39].

In contrast, the transient current of the NWs annealed in N_2_ is not blocked at any bias, which indicates an almost complete vanishing of both Schottky barriers (green line, Figure 6b). Only at very low, negative fields up to roughly −2 kV/cm, we found a Fowler–Nordheim tunneling behavior for the electrons passing from the PtIr tip to the TiO_2_ NW (Figure 6c). For larger field amplitudes, the transient current becomes linear, showing a relatively large ohmic resistance (green line, Figure 6a). However, the slope and thus the absolute ohmic resistance depends on the applied voltage.

## 3. Discussion

Our results show that different heat treatments change the nanostructure and the properties of hydrothermally grown rutile TiO_2_ NWs significantly. In the following, the interaction of the structural changes on the properties will be discussed.

As-grown NWs are intensively studied and used in many application and thus serve as a reference in this work. The detailed electron microscopic analysis showed that these NWs are rutile TiO_2_, but contrary to many reports [40], they are not single-crystalline [30,32]. Instead, they show a meso-crystalline structure that is built by a bundle of nanofibers and incorporate many crystal defects [30], especially a high amount of O_vac_ [32]. The optical band gap of around 3 eV is in accordance with literature values for rutile TiO_2_ [2], but the Urbach energy of 0.61 eV is much larger than reported for single-crystalline rutile TiO_2_ nanoparticles [41], and can be assigned to the high defect disorder of the O_vac_ and other structural defects. In addition, the O_vac_ influences the electronic properties, as they are prominent electron donors that tune TiO_2_ into an n-type semiconductor [42,43,44]. The O_vac_ in as-grown TiO_2_ NWs have two effects on the electronic properties. In the first instance, the local donor density moves the Fermi level upward, closer to the conduction band minimum. As a consequence, the summit of the Schottky barrier between the metallic cathode and the TiO_2_ drops with increasing electron donor density close to the interface. In addition, an increased number of O_vac_ lowers the resistivity of TiO_2_ by increasing the number of mobile electrons in the conduction band [45,46] and thus the as-grown rutile TiO_2_ NWs show n-type conductivity.

As shown in a previous study, TiO_2_ NWs annealed in air have a significantly reduced density of O_vac_ in the crystal structure, as vacancy condensation takes place during the heat treatment [32]. The NWs are single-crystalline and the rutile crystal structure of the NWs annealed in air is almost O_vac_ free. In addition, the vacancies close to the NW surface are vanished due to the oxygen atmosphere during the heat treatment, resulting in NWs that have only a 1.8 nm thick surface layer, which deviates from the perfect rutile TiO_2_ environment, as shown by changes in the ELNES. These changes during the heat treatment influence the optical and electronic properties of the NW array, as both the amount of trap states and electron donors incorporated in the crystal structure are significantly reduced. This deduction is verified by the UV-Vis measurements, which show that the band gap as well as the Urbach energy shrink. The reduced band gap can be assigned to less O_vac_ in the crystalline rutile TiO_2_ [47] and a high Ti^3+^ concentration in the defective area surrounding each void [32]. It is noteworthy that these NWs appear white although the band gap indicates absorption in the visible blue regime. This effect is related to a strong light scattering, which is caused by the high refractive index of TiO_2_ [48]. Furthermore, a reduced Urbach energy indicates less disorder. However, Urbach energy is still higher than expected for a single-crystalline rutile nanoparticle [41]. This deviation results from the 1.8 nm thick surface layer covering the NWs and a defective, Ti^3+^ rich area surrounding each void [32]. Concurrently, the transient current is blocked over a broad range of electrical fields. Only for highly negative electrical fields a Schottky emission-limited current can be detected. These results are in good accordance with the discussion above. As the density of O_vac_ is significantly reduced in the rutile crystal structure of NWs annealed in air, the Schottky barrier heights and the bulk resistance are expected to increase. Nevertheless, at high negative electrical fields the Schottky barrier can still be passed. We assume a constant work function for the PtIr tip and the FTO substrate for all experiments, so the Schottky barrier is mainly influenced by the Fermi level of the TiO_2_ NWs. Structural inhomogeneity at the TiO_2_ NW tip surface might influence the Schottky barrier, but SEM analysis showed that the surface of the NWs annealed in air is the smoothest, so we assume only a minor contribution of surface inhomogeneity on the height of the Schottky barrier.

Annealing in N_2_ changes the distribution of O_vac_ as well. According to the TEM results presented in this work, the NWs annealed in N_2_ have a complex core-shell structure. From these results, it is reasonable to assume that the defect density in the core, which is riddled by voids, is similar to the defect density of the NWs annealed in air. Consequently, the electronic properties of the core, possessing a low defect density, are similar to the electronic properties of NWs annealed in air. However, the IV-characteristics measured for the NWs annealed in N_2_ differ strongly from those, measured for the NWs annealed in air. Thus, the core of the NW annealed in N_2_ has no significant influence on the conductivity in these NWs. The Fowler–Nordheim tunneling behavior that occurs at low electrical fields is supposed to be a result of a disordered surface layer (Figure 4a,c) covering the metal-like TiO_2−*x*_ shell. For strong electric fields, the influence of this ultra-thin layer is negligible. Without this metallization, the Schottky barrier is much thicker and Schottky emission, as observed for the as-grown NWs, instead of tunneling dominates. The metal-like behavior of the shell is in good agreement with the black color of the NW array, as absorption throughout the entire spectral range is common for metals. Several observations indicate that the metallization takes place in a confined volume. Firstly, the optical measurements are still dominated by the properties known for white TiO_2_. It is known that the transmittance of light of thin metal films drops below 20% for films being thicker than about 10–20 nm [49]. As our NW arrays show a high transmittance, it is reasonable to assume that the metallic part in the NWs annealed in N_2_ does not exceed 20 nm. In addition, the ohmic resistance measured for these NWs is relatively large. Such large ohmic resistances stem from the tiny cross-sections of the highly conductive part of the NWs annealed in N_2_. According to the TEM and EELS results, NWs annealed in N_2_ are covered by a TiO_2−*x*_ shell that contains a very high amount of O_vac_, as the vacancies cannot be removed at the surface due to the slightly reducing environment of the N_2_ atmosphere. Although an incorporation of N cannot be ruled out completely due to the EELS detection limit of around 1%, we assume no influence of a potential N doping (which would be below 1 at% of N) on the electrical properties. This assumption is based on the fact that changes in the electronic properties for TiO_2−2*x*_N*_x_* were only detected for N incorporation higher than 5 at% N [50]. Such high concentrations can be excluded due to the absence of an N K-edge in the EEL spectra throughout the NW, although it is not possible to confirm this result with other methods due to signal overlap. Nevertheless, even undoped but strongly reduced TiO_2−*x*_, as found in the shell of the NW annealed in N_2_, is highly conductive [26,44]. Hence, it is reasonable to assume that the TiO_2−*x*_ shell is responsible for the unusual properties of these NWs, but due to the small dimensions it is difficult to localize the origin of these effects within the shell. According to the TEM results, the shell can be divided in three parts, namely the disordered surface layer (2.2 nm), the crystalline TiO_2−*x*_ shell (8–20 nm) and a defective interfacial area between the TiO_2−*x*_ shell and the TiO_2_ core (1.9 nm). The high Urbach energy measured for these NWs originates from the high degree of disorder in the surface layer. Similar surface layers were found in various black TiO_2_ nanomaterials and seem to be the origin of the black color [16]. This change in color is mainly related to the presence of a big Urbach tail at the upper part of the valence band [16]. These results are in good accordance with the high Urbach energy which was measured for NWs annealed in N_2_. The metallization and the high transient current might result from the entire shell but there are some indications that it is confined on the defective interface between the TiO_2−*x*_ shell and the TiO_2_ core. The EELS analysis showed a higher concentration of Ti^3+^ at this interface, which might arise from a great amount of O_vac_ confined at this interface. Both are electron donor type defects and can lead to high conductivity. Lü et al. found a similar conducting interface at the homojunction of a bilayer thin film. This homojunction is formed between an oxygen-deficient, amorphous TiO_2−*x*_ layer with around 20 nm thickness and a comparable thick layer of anatase TiO_2_ [29]. Our experimental setup does not allow direct proof of this assumption, but the results obtained in this study give evidence that not the entire shell, but a conductive interface might be responsible for the highly conducting properties of the black NWs annealed in N_2_. In addition, there is a certain hysteresis of the IV characteristics, which indicates that the O_vac_ are able to drift through the TiO_2−*x*_ shell. This effect is well known from resistive switching [51,52,53] and might be the reason why the O_vac_ cannot be detected by annular bright field STEM. Due to the high mobility of the O_vac_, their density at the PtIr/TiO_2_ and FTO/TiO_2_ interfaces differ slightly, resulting in the observed asymmetry of the IV characteristics for positive and negative applied bias.

## 4. Materials and Methods

### 4.1. Synthesis Procedure

TiO_2_ NW arrays were synthesized by a hydrothermal procedure adapted from Liu et al. [40]. All chemicals were used as supplied without further purification. In a typical synthesis, 250 μL titanium butoxide (Ti(*n*OBu)_4_), Sigma-Aldrich, St. Louis, MI, USA) was dropped into a mixture of 5 mL concentrated hydrochloric acid (37 wt%, analytical grade, Sigma-Aldrich) and 5 mL deionized water under vigorous stirring. Ultrasonically cleaned (isopropyl alcohol, acetone, ethanol) FTO substrates were placed vertically in a Teflon liner, which was filled with the growth solution and placed into a steel autoclave. The hydrothermal reaction was performed at 150 °C for 4.5 h. Afterwards, the autoclave was cooled down to room temperature. The FTO substrates, covered with TiO_2_ NW arrays, were rinsed with deionized water and dried with compressed air. Heat treatment of the samples was performed at 500 °C (50 °C/min ramp up to 500 °C) on an Anton Paar DHS 1100 (Anton Paar, Graz, Austria) heating stage. For the TiO_2_ NWs annealed in N_2_, a constant N_2_ atmosphere of 1.35 bar was applied during the experiment, whereas the other sample was annealed in air.

### 4.2. Characterization

SEM analysis: The morphology of the NW arrays in top-view was investigated using a Zeiss AURIGA Modular CrossBeam workstation (Zeiss, Oberkochen, Germany) equipped with an in-lens detector. All measurements were carried out at 4 kV.

TEM analysis**:** TEM was applied for the morphological and crystallographic analysis. The TiO_2_ nanowires were scraped off the FTO substrate and the resulting powder was dispersed on a copper grid with a holy carbon film. A Philipps CM20 (FEI, Hillsboro, OR, USA) and a Jeol JEM-2200FS field emission gun instrument (Jeol, Akishima, Japan), both operated at 200 kV, were used for conventional bright field TEM, selected area electron diffraction, and HR TEM.

STEM images and EELS data were acquired at 300 kV with a FEI Titan Themis 60–300 (FEI, Hillsboro, OR, USA) equipped with a high brightness field emission (XFEG™) source, a monochromator, an aberration-corrector for the probe-forming lens system, a BRUKER EDS Super X detector, and a high-resolution energy filter (post-column Quantum ERS energy filter). EEL spectra were acquired in STEM mode with a dispersion of 0.1 eV per channel. To measure the band gap on a local scale, low-loss spectra in monochromated STEM mode were acquired with a dispersion of 0.01 eV. An energy resolution of 0.3 eV, as determined by the full-width at half maximum of the zero-loss peak, was obtained. Using a power-law fit, the tail of the zero-loss peak was removed and the band gap was extracted according to the linear fit method [54]. For all EELS measurements, the convergence semi angle was 23.8 mrad and the collection semi-angle was 35 mrad. All EELS data were taken using the dual-channel acquisition technique [55] and the spectra were corrected for dark current and channel-to-channel gain variation [56]. The background was removed using a standard power law fit [56].

EEL spectra of the Ti-L_2,3_ edge were used to determine the Ti^3+^/Ti^4+^ ratio with high lateral resolution. Therefore, a calibration technique of Stoyanov et al. [34]. was used. It is based on the position and intensities of the Ti L_2_ and L_3_ white lines.

UV-Vis spectroscopy: A PerkinElmer Lambda 800 spectrometer (PerkinElmer, Waltham, MA, USA) in transmission mode was utilized to measure the absorption spectra in a wavelength range of 350–850 nm. The step size was 1 nm. The detected UV–Vis data were used to determine direct and indirect band gaps using Tauc plots [57] and to calculate the Urbach energy [38] of the different samples.

IV characteristics: Qualitative information about the electronic properties of the investigated TiO_2_ NWs was obtained by IV-measurements. A platinum-iridium (PtIr) (4:1) tip served as a top electrode. The IV-characteristics of the transient current through the PtIr/TiO_2_/FTO sandwich were determined. The tip was taken because typical deposition techniques used for flat metal electrodes would infiltrate the interspace between the NWs and cause shorts. The tip was placed manually and softly on the NW array and pushed by its own weight (0.1 g) on a bunch of NWs during the measurement. The bottom contact was established by removing the NWs using a diamond writer and connecting the uncovered FTO with a thin insulated copper wire and a drop of silver paste. The sample holder was transferred into a vacuum chamber, where the humid air was replaced by dry nitrogen during several pumping and purging steps. A Keithley 2401 (Ketihly Instruments, Cleveland, OH, USA) was used as a voltage source and to measure the transient current. In the presented graphic, a positive electric field is pointing from the PtIr tip towards the FTO and the IV-curves were obtained by changing the field from negative to positive values.

## 5. Conclusions

In this study, we propose heat treatments in different environments in order to manipulate the structure of hydrothermally grown rutile TiO_2_ NWs in such a way that their optical and electrical properties can be tailored. The as-grown NWs incorporate a high amount of defects, especially O_vac_, which are responsible for the n-type conductivity in these NWs. Independent of the environment, the heat treatment leads to a condensation of these vacancies and to the formation of single-crystalline, lattice defect free, rutile TiO_2_ NWs that incorporate voids. The absence of O_vac_ results in a blocking of the transient current and concurrently improves the optical properties by decreasing the band gap and Urbach energy. For an oxidizing environment, such as air, the resulting NWs are almost insulating. Although NWs annealed in N_2_ contain up to around 80% of an insulating rutile TiO_2_ core, their properties are completely different. They possess a black color and an almost metal-like conductivity. These properties are related to the slightly reducing atmosphere of N_2_ during the heat treatment. It inhibits the vanishing of the surface-near O_vac_ and thus a core-shell structure with a highly oxygen deficient shell is formed.
